# The Effects of Regular Exercise on Circulating Cardiovascular-related MicroRNAs

**DOI:** 10.1038/s41598-019-43978-x

**Published:** 2019-05-17

**Authors:** Jacob L. Barber, Kia N. Zellars, Kurt G. Barringhaus, Claude Bouchard, Francis G. Spinale, Mark A. Sarzynski

**Affiliations:** 1 Department of Exercise Science, University of South Carolina, Columbia, SC USA; 2 Cardiovascular Translational Research Center, University of South Carolina School of Medicine and WJB Dorn Veteran Affairs Medical Center, Columbia, SC USA; 3 Human Genomics Laboratory, Pennington Biomedical Research Center, Baton Rouge, LA USA

**Keywords:** Risk factors, Predictive markers, Cardiovascular genetics

## Abstract

The purpose of the present study was to examine the effects of regular exercise on the abundance of targeted circulating microRNAs (miRNAs). The present analysis examined 20 previously sedentary adults from the HERITAGE Family Study who completed 20 weeks of endurance exercise training. The expression of 53 miRNAs related to cardiovascular disease were measured in serum collected at baseline and post-training by performing RT-qPCR on the Human Cardiovascular Disease miRNA array (Qiagen, Germany). The effect of regular exercise on circulating miRNAs was assessed using paired t-tests of baseline and post-training expression levels. A false discovery rate threshold of 5% was used to determine significance. Regular exercise resulted in significantly decreased mean serum expression of nine miRNAs (miR-486-5p, let-7b-5p, miR-29c-3p, let-7e-5p, miR-93-5p, miR-7-5p, miR-25-3p, miR-92a-3p, and miR-29b-3p; fold change range: 0.64–83, p = 0.0002–0.01) and increased mean expression of five miRNAs (miR-142-3p, miR-221-3p, miR-126-3p, miR-146a-5p, and miR-27b-3p; fold change range: 1.41–3.60, p = 0.001–0.006). Enrichment analysis found that these 14 miRNAs target genes related to over 345 different biological pathways. These results provide further evidence of the effects of regular exercise on the circulating miRNA profile.

## Introduction

MicroRNAs (miRNAs) are small non-coding regulatory RNAs that participate in the regulation of gene expression via post-transcriptional modification of messenger RNA (mRNA)^1,2^. MiRNAs silence mRNAs through two distinct mechanisms: mRNA degradation or repression of mRNA translation^2^. Over 2,500 different miRNAs have been identified in humans and despite accounting for an estimated 1–5% of the human genome, miRNAs are estimated to regulate greater than 30% of protein coding genes^2–4^. MiRNAs are transported into circulation within exosomes, protein complexes, or microvesicles^2,5^. The transport of miRNAs in these complexes prevents their breakdown and allows miRNAs to circulate and act on target cells throughout the body^1,2^. Thus, circulating miRNAs represent novel biomarkers for diseases such as cancer and cardiovascular disease (CVD)^2,6,7^.

Altered circulating miRNA profiles have been identified in patients with various diseases, including different forms of cancer, diabetes, and CVD^8,9^. Additionally, Zampetaki *et al*^10^. found that baseline miRNA profiles were associated with incident myocardial infarction over ten years in an elderly cohort, suggesting that miRNAs can also be used to predict future disease. MiRNAs have also been functionally related with multiple steps in the progression towards atherosclerosis and associated with acute myocardial infarction, coronary artery disease, and unstable angina^6,11^. Additionally, miRNAs play an essential role in many biological processes such as angiogenesis, mitochondrial metabolism, and cardiac/skeletal muscle hypertrophy^12–14^.

The associations of miRNAs with disease, as well as the physiological effects of miRNAs on multiple biological pathways, demonstrate the potential clinical relevance of circulating miRNAs as biomarkers of disease. Therefore, identifying treatments and interventions to alter the circulating miRNA profile are of interest. Exercise represents one such intervention, as exercise has repeatedly been shown to lower CVD, cancer, and mortality risk^15–17^. However, the effects of exercise on the circulating miRNA profile are not completely understood. The current literature shows an effect of acute exercise on the circulating miRNA profile, suggesting that miRNAs play a role in physiological adaptations to acute exercise^18,19^. However, less is known about the effects of regular exercise on the circulating miRNA profile. The current literature examining regular exercise and circulating miRNAs is somewhat limited in that many studies have examined fewer than 10 targeted miRNAs, with little overlap of miRNAs between studies^19^. Furthermore, little is known about how exercise affects miRNAs related to CVD. Therefore, the purpose of the current study was to explore the potential effects of regular exercise on a relatively large panel of miRNAs known to be associated with CVD. Specifically, the Human Cardiovascular Disease miRNA array (Qiagen, Germany) was examined in 20 individuals from the HERITAGE Family Study. This array includes 84 miRNAs that are differentially regulated during CVD progression and have been associated with CVD in multiple studies involving a variety of CVD models and a variety of miRNA profiling methodologies^20–28^. We hypothesized that regular exercise would significantly alter the circulating miRNAs related to CVD.

## Results

**Figure 1:**
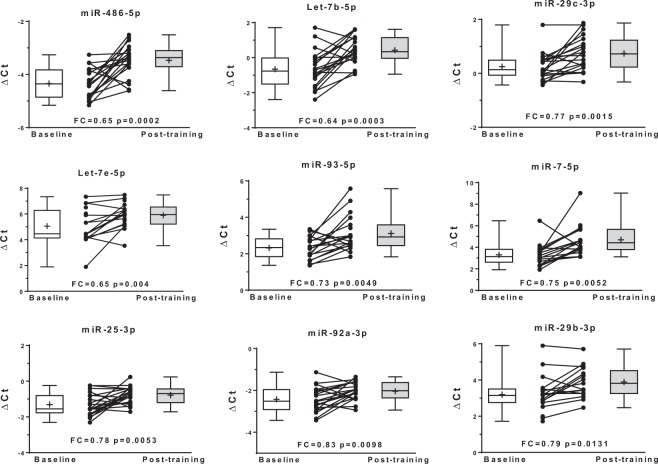
Baseline and post-training normalized cycle threshold values (ΔCt) along with individual exercise response for nine miRNAs with decreased circulating expression (higher ΔCt) following regular exercise. FC, fold change.

**Figure 2:**
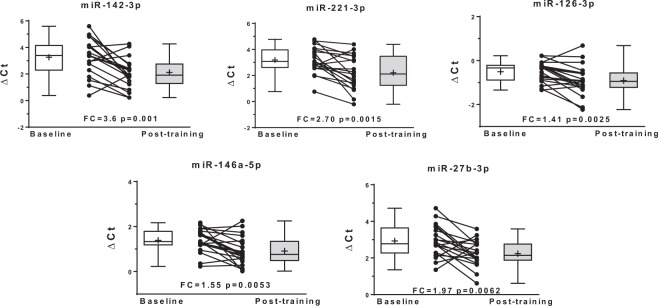
Baseline and post-training normalized cycle threshold values (ΔCt) along with individual exercise response for five miRNAs with increased circulating expression (lower ΔCt) following regular exercise. FC, fold change.

Baseline characteristics including mean values for the standard lipid panel and other cardiometabolic risk factors are shown in Table 1. No significant differences at baseline were found between sexes for these traits. The circulating expression levels of 14 miRNAs were significantly altered with regular exercise (FDR q < 0.05). Regular exercise resulted in decreased expression of nine miRNAs (miR-486-5p, let-7b-5p, miR-29c-3p, let-7e-5p, miR-93-5p, miR-7-5p, miR-25-3p, miR-92a-3p, and miR-29b-3p) (Fig. 1) and increased expression of five miRNAs (miR-142-3p, miR-221-3p, miR-126-3p, miR-146a-5p, and miR-27b-3p) (Fig. 2 and Table 2). These 14 miRNAs were entered into pathway/target analysis and combined are predicted to target over 7,500 genes, while individually each miRNA contributes to the regulation of many genes (range: 57–1406) (Supplemental Table S1**)**. Connectivity analysis of the 14 miRNAs and their gene targets showed the minimally connected network was comprised of 47 genes and 127 edges (Supplemental Fig. S1). Enrichment analysis of the 14 miRNAs showed they were associated with over 345 different biological pathways, including PI3K-Akt, VEGFA-VEGFR2, insulin, and leptin signaling pathways (Table 3).

**Table 1:** Baseline characteristics of participants (n = 20).

Variable	Mean (SD)
Sex (Male/Female)	(10/10)
Age (years)	43.7 (12.8)
BMI (kg/m^2^)	26.2 (3.7)
VO_2_max (mL/kg/min)	28.8 (6.9)
Waist circumference (cm)	90.2 (11.4)
Percent fat	28.1 (7.8)
SBP (mmHg)	116.6 (12.4)
DBP (mmHg)	65.9 (9.7)
HDL-C (mg/dL)	43.9 (10.6)
LDL-C (mg/dL)	135.4 (33.6)
TC (mg/dL)	204.4 (36.7)
TG (mg/dL)	156.2 (83.0)
CRP (mg/dL)	0.29 (0.3)

BMI: body mass index, HDL-C: high-density lipoprotein cholesterol, LDL-C: low-density lipoprotein cholesterol, TC: total cholesterol, TG: triglycerides, CRP: C-reactive protein, SBP: systolic blood pressure, DBP: diastolic blood pressure, VO_2_max: maximum rate of oxygen consumption.

**Table 2:** The effect of regular exercise on circulating microRNAs related to cardiovascular disease.

miRNA	n	Fold-Change (SD)	*P value* ^a^	*q value* ^b^
miR-486-5p	20	0.65 (0.4)	**0**.**0002**	0.0009
let-7b-5p	20	0.64 (0.5)	**0**.**0003**	0.0019
miR-142-3p	19	3.60 (3.8)	**0**.**001**	0.0028
miR-221-3p	19	2.70 (2.6)	**0**.**0015**	0.0038
miR-29c-3p	20	0.77 (0.3)	**0**.**0015**	0.0047
miR-126-3p	20	1.41 (0.5)	**0**.**0025**	0.0057
let-7e-5p	18	0.65 (0.4)	**0**.**004**	0.0066
miR-93-5p	18	0.73 (0.5)	**0**.**0049**	0.0075
miR-7-5p	16	0.75 (1.3)	**0**.**0052**	0.0085
miR-146a-5p	20	1.55 (0.8)	**0**.**0053**	0.0094
miR-25-3p	20	0.78 (0.4)	**0**.**0053**	0.0104
miR-27b-3p	20	1.97 (1.2)	**0**.**0062**	0.0113
miR-92a-3p	20	0.83 (0.4)	**0**.**0098**	0.0123
miR-29b-3p	16	0.79 (0.4)	**0**.**0131**	0.0132
let-7d-5p	20	0.84 (0.4)	0.0175	0.0142
miR-17-5p	16	2.04 (1.7)	0.0208	0.0151
miR-424-5p	19	1.85 (1.5)	0.0439	0.016
miR-23a-3p	20	1.35 (0.6)	0.0477	0.017
miR-222-3p	18	1.36 (0.6)	0.0483	0.0179
miR-30c-5p	20	1.62 (0.9)	0.0579	0.0189
miR-27a-3p	20	2.04 (1.6)	0.0613	0.0198
miR-103a-3p	13	7.42 (12.3)	0.079	0.0208
let-7a-5p	20	1.06 (1.1)	0.0875	0.0217
miR-29a-3p	20	1.50 (1.0)	0.0907	0.0226
miR-24-3p	20	1.47 (0.7)	0.0917	0.0236
miR-125a-5p	20	1.46 (0.9)	0.1006	0.0245
miR-16-5p	20	1.01 (1.0)	0.1025	0.0255
let-7c	20	1.11 (1.2)	0.1324	0.0264
miR-342-3p	20	1.47 (1.0)	0.1514	0.0274
miR-15b-5p	20	0.95 (0.4)	0.1564	0.0283
miR-181b-5p	14	1.67 (1.4)	0.2046	0.0292
miR-224-5p	10	2.37 (2.2)	0.2192	0.0302
miR-125b-5p	15	2.57 (2.9)	0.2577	0.0311
miR-130a-3p	11	2.20 (1.7)	0.3041	0.0321
miR-195-5p	20	1.48 (2.5)	0.3096	0.033
miR-185-5p	18	1.60 (2.1)	0.3166	0.034
miR-10b-5p	14	3.42 (4.3)	0.3411	0.0349
miR-150-5p	20	1.40 (0.8)	0.3693	0.0358
miR-23b-3p	19	1.34 (0.6)	0.4056	0.0368
miR-223-3p	20	1.37 (1.3)	0.4779	0.0377
miR-320a	20	1.13 (1.0)	0.5106	0.0387
miR-30e-5p	18	1.72 (2.1)	0.6095	0.0396
miR-423-3p	13	2.21 (2.3)	0.6245	0.0406
miR-98-5p	18	1.31 (0.9)	0.6554	0.0415
miR-26a-5p	20	1.11 (0.5)	0.7655	0.0425
miR-122-5p	20	1.41 (1.4)	0.7881	0.0434
miR-26b-5p	19	3.97 (13.1)	0.82	0.0443
miR-451a	20	1.89 (2.9)	0.8475	0.0453
miR-100-5p	19	1.59 (1.6)	0.8936	0.0462
miR-30d-5p	20	1.30 (1.1)	0.9146	0.0472
miR-30a-5p	20	1.31 (1.0)	0.9536	0.0481
miR-21-5p	20	1.06 (0.4)	0.9546	0.0491
miR-365a-3p	15	1.65 (1.8)	0.9907	0.05

n represents the number of subjects in which the miRNA was detectable in both baseline and post-training samples.

^a^*P* values are the result of paired t-tests of normalized baseline and post-training miRNA expression levels.

^b^When *P* < *q* the change in expression with training is deemed significant, which is indicated in bold.

**Table 3:** Select pathways associated with the 14 microRNAs significantly altered in response to regular exercise and the number of genes per pathway targeted by these microRNAs.

Pathway	Number of miRNAs Involved	Genes Targeted
PI3K-Akt Signaling	$14$	$123$
PI3K-Akt-mTOR Signaling	$14$	$103$
VEGFA-VEGFR2 Signaling	$14$	$77$
Insulin Signaling	$14$	$53$
Leptin Signaling	$14$	$48$

Pathways were identified from the exRNA atlas database (http://exrna-atlas.org).

## Discussion

This exploratory study found that regular exercise significantly altered the circulating expression levels of 14 miRNAs related to CVD in 20 previously sedentary, but otherwise healthy adults from the HERITAGE Family Study. The 14 miRNAs altered with exercise potentially contribute to the regulation of thousands of genes and are associated with hundreds of different biological pathways. Thus, the potential downstream biological effects of altered miRNA levels with regular exercise are enormous.

Little is known about the potential mechanism(s) by which exercise could alter circulating miRNA expression. MiRNA expression can certainly be regulated and altered, but the regulation of miRNAs is complex and not fully understood^29^. One potential mechanism could be an augmented degradation of the miRNAs in circulation with exercise. However, miRNAs are transported within vesicles or within protein complexes that prevent degradation^1,2^, thus this seems unlikely. Another potential mechanism could be the promotion of selective uptake of these miRNAs by skeletal muscle with exercise, as several studies have demonstrated the ability of target cells, such as skeletal muscle, to take up circulating miRNAs^1,2,30^. Similarly, it has been proposed that exercise may cause the release of certain miRNAs from skeletal muscle, either through muscle damage or selective release in response to acute or regular exercise^31,32^. Thus, one plausible explanation is that the increased or decreased expression of these 14 miRNAs in circulation with regular exercise may be due in part to increased uptake of miRNAs by skeletal muscle (9 of 14 decreased with regular exercise) and the converse increased release of certain miRNAs by skeletal muscle (5 of 14 increased with regular exercise). Baggish *et al*^33^. found that regular exercise resulted in elevated levels of circulating miR-222, miR-21, and miR-221. Interestingly, expression levels of these same three miRNAs were also elevated following acute exercise, indicating a potential additive effect of repeated acute bouts, as with training, that serves to increase circulating expression of certain miRNAs^33^.

Previous studies have examined the response to exercise of some of the same miRNAs included in this study. We found that regular exercise significantly decreased the expression of circulating miR-486-5p. Similarly, a study of 11 men found a single cycling exercise session and four weeks of regular exercise both significantly decreased circulating levels of miR-486-5p^31^. A 90-day period of rowing training in 10 males increased circulating miR-221-3p and miR-146a-5p expression^33^, the same responses found with cycle ergometer training in the current study. Circulating miR-92a-3p and miR-29b-3p expression levels were both significantly decreased in seven males following 12 weeks of regular exercise on cycle ergometers^34^. Both of these miRNAs also significantly decreased with training in the present analysis. The agreement with the current literature on the effects of regular exercise on these select miRNAs is encouraging and strengthens the existing evidence of a significant effect of exercise on the circulating miRNA profile. However, many of the 14 circulating miRNAs significantly altered with regular exercise in the current study have not been previously examined.

Our study benefitted from the use of a miRNA PCR array that included 84 miRNAs known to be associated with CVD. Additionally, the entire assay from RNA extraction to miRNA quantification was done using a fully robotic automated platform (QIAcube workstation) minimizing any potential pipetting errors. The study also benefitted from a fully standardized and supervised exercise program, thus all included subjects received the same relative dose of exercise and fully adhered to the program. Further research is also needed to determine the mechanisms by which exercise alters circulating miRNA expression and the association of changes in the circulating miRNA profile with changes in clinical outcomes. Importantly, the present study must be seen as a small, exploratory study. The same research questions should be investigated in greater detail with a much larger sample size and an untargeted approach to accurately and comprehensively examine the effects of regular exercise on the circulating miRNA profile. This study was also limited by a lack of technical replicates in sample analysis. Finally, further research in the field of circulating miRNAs is needed to create clear standards for data handling. Specifically, the difficulty in differentiating between missing data due to a true lack of expression in the sample and missing data due to various experimental issues is a major limitation in the field. Current conventions suggest ignoring Ct values above detection threshold limits, however these ‘missing’ data may have physiological relevance as the appearance or disappearance of a miRNA in response to an intervention is potentially even more meaningful than changes in expression. Similarly, further research is needed to determine an ideal normalization strategy for circulating miRNAs, as different normalization methodologies may lead to different conclusions and impact reproducibility and comparability of studies.

In summary, regular exercise may alter the circulating miRNA profile of select CVD related miRNAs and these alterations may have physiological relevance given the number of genes regulated by and diverse roles of these miRNAs. The current study provides further support for the effects of regular exercise on circulating miRNAs miR-486-5p, miR-146a-5p, miR-92a-3p, miR-29b-3p, and miR-221-3p and new evidence for other miRNAs. The existing evidence suggests that circulating miRNAs should be thoroughly investigated as biomarkers of regular exercise response. Further research is needed on the effects of exercise on the global circulating miRNA profile to potentially identify novel miRNAs associated with exercise and confirm the existing findings.

## Materials and Methods

The HERITAGE Family Study was designed to examine the role of genetic factors on cardiometabolic responses to endurance regular exercise. The HERITAGE cohort is composed of 481 whites (232 men and 249 women) from 99 families and 250 blacks (88 men and 162 women) from 105 family units that completed a 20-week endurance exercise program at one of four clinical centers (Indiana, Minnesota, Québec, Texas). For the present analysis, 20 non-related white subjects (10 males, 10 females) from the Québec center that completed the 20-week exercise program were selected as part of an ongoing ancillary study. The study design, training protocol, and measurements have been described in detail elsewhere^35^. Briefly, the training program consisted of three weekly exercise sessions on cycle ergometers starting at the heart rate associated with 55% VO_2_max and progressing to the heart rate associated with 75% baseline VO_2_max for the final six weeks of the study. The study protocol was approved by the Institutional Review Boards at each of the five participating centers of the HERITAGE Family Study consortium (Indiana University, Laval University, University of Minnesota, Texas A&M University, and Washington University at St. Louis). Written informed consent was obtained from each study participant. All research was performed in accordance with the Declaration of Helsinki.

### Exercise tests

Sub-maximal and maximal exercise tests on a stationary cycle ergometer were administered prior to training and additional tests were given following the completion of training^35^. Maximal cycle ergometer tests were performed twice on separate days at least 48 hours apart. VO_2_max was determined by the average of the two tests if the values were within 5% of each other, or the higher of the two values if the measures differed by more than 5%^36^.

### Blood collection

Blood samples were obtained in the morning after a 12 hour fast, both at baseline and again 24 hours after the last training session. Serum was collected using standard methods^36^.

### RNA extraction

Total RNA was extracted on the QIAcube workstation from baseline and post-training whole serum samples using miRNeasy Serum/Plasma Advanced Kits (Qiagen, Hilden, Germany). Samples were spiked with a known amount of synthetic C. elegans miR-39-3p (cel-miR-39-3p).

### MiRNA quantification

**Figure 3:**
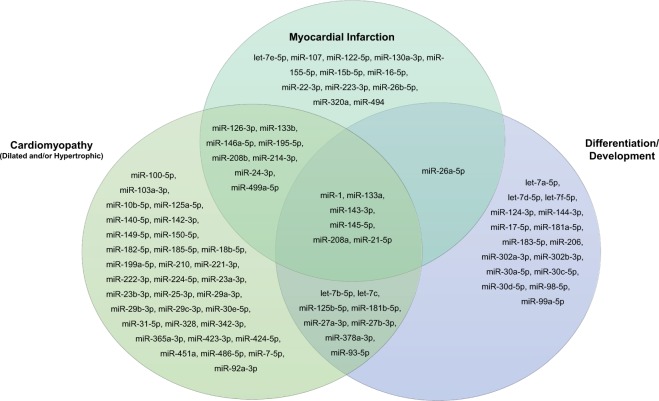
The 84 miRNA sequences on the Human Cardiovascular Disease miScript miRNA PCR Array grouped by their functional domains.

MiRNA quantification was performed using reverse transcription real-time polymerase chain reaction (RT-qPCR). Briefly, cDNA was transcribed from the extracted RNA and then aliquoted into the Human Cardiovascular Disease miScript miRNA PCR Array (Qiagen, Hilden, Germany). This array contains primers for 84 miRNA sequences, an individual miRNA primer sequence per well, identified as exhibiting altered expression during cardiovascular disease. The selected miRNAs are listed in Fig. 3. All cDNA steps and PCR setup were performed by the QIAgility instrument (Qiagen, Hilden, Germany) using an automated pipetting protocol. RT-qPCR was performed on the miRNA PCR array in the Rotor-Disc 100 format by the Rotor-Gene Q real-time PCR cycler (Qiagen, Hilden, Germany). Rotor-Gene PCR cycling was performed according to the manufacturer’s suggested protocol and conditions. Cycle threshold (Ct) represents the cycle number at which there is an exponential increase in miRNA fluorescence. Individual miRNAs were determined to be detected when Ct values were lower than 33, while miRNAs with a Ct value ≥ 33 were considered not detected. Only miRNAs detected in at least 50% of subjects were retained for analysis, resulting in 53 total miRNAs analyzed.

### MiRNA validation

To validate the miRNA quantification methods, samples from six participants were randomly selected from the study inventory for analysis. Total RNA was isolated and spiked with a known amount of synthetic cel-miR-39-3p and miRNA analyzed as described above. Analysis demonstrated that cel-miR-39-3p exhibited excellent linearity between the target input and measured values with regression coefficients approaching 1 and the qPCR-based extraction efficiency (Efficiency = 10^(−1/slope)^−1) was approximately 80% (79.08% ± 11.26%). Additionally, as a positive control for RT-qPCR performed with the Human Cardiovascular Disease miScript miRNA PCR Array, three wells of the array contained cel-miR-39-3p. Using this internal control, the Ct coefficient of variation (CV) values were determined to be 1.14% ± 0.49% intraplate and 0.9% ± 0.62% interplate. Using these automated methods and repeating this miRNA array in triplicate for these six samples, the overall CV for each miRNA was less than 20%.

### MiRNA normalization and fold change calculation

Normalized miRNA expression at baseline and post-training are represented by ΔCt. ΔCt values were calculated by subtracting the global geometric mean signal of miRNAs that were commonly expressed in the present study from individual miRNA Ct values as previously described^37,38^. Commonly expressed miRNAs were identified as those with Ct values < 33 in all samples, utilizing the GeneGlobe Data Analysis Center PCR software (Qiagen). ΔΔCt was then calculated by subtracting baseline ΔCt values from post-training ΔCt values. Fold change was calculated as 2^−∆∆Ct^.

### Statistical analysis

The effects of regular exercise on circulating miRNAs were assessed using paired t-tests of baseline and post-training ΔCt levels. A false discovery rate (FDR) threshold of 5% using the Benjamini-Hochberg procedure was implemented to define statistical significance^39^. All analyses were performed using SAS 9.4 (Cary, NC).

### MiRNA target prediction and pathway analysis

Experimentally validated miRNA targets were identified using miRNet (https://www.mirnet.ca), which utilizes data from well-annotated databases: miRTarBase v7.0, TarBase V7.0, and miRecords^40,41^. The connectivity of the 14 miRNAs and their target genes was visualized using miRNet with a minimum network adjustment to simplify the network visualization. Pathway analysis was performed using the extracellular RNA Atlas (http://exrna-atlas.org)^42^. All 14 miRNAs significantly altered with regular exercise were entered into the pathway analysis.

## Supplementary information

Supplemental Material

## Data Availability

The datasets generated during and/or analyzed during the current study are available from the corresponding author on reasonable request.
