# Supplementary material for: The Effects of Regular Exercise on Circulating Cardiovascular-related MicroRNAs
Source: Sci Rep. 2019 May 17;9:7527. doi: 10.1038/s41598-019-43978-x (PMC6525243; doi:10.1038/s41598-019-43978-x)
Supplement: Supplementary file 1 — Supplemental Material [file 41598_2019_43978_MOESM1_ESM.docx]

**The Effects of Regular Exercise on Circulating Cardiovascular-related MicroRNAs SUPPLEMENTAL MATERIAL**

Jacob L. Barber^1^, Kia N. Zellars^2^, Kurt G. Barringhaus^2^, Claude Bouchard^3^, Francis G. Spinale^2^, Mark A. Sarzynski^1^.

^1^Department of Exercise Science, University of South Carolina, Columbia, SC

^2^Cardiovascular Translational Research Center, University of South Carolina School of Medicine and WJB Dorn Veteran Affairs Medical Center, Columbia, SC

^3^Human Genomics Laboratory, Pennington Biomedical Research Center, Baton Rouge, LA

**Corresponding Author:**

Mark A. Sarzynski

Department of Exercise Science, University of South Carolina

921 Assembly Street, Room 301 Columbia, SC 29201

Phone: (803) 777-9510

Email: [sarz@mailbox.sc.edu](mailto:sarz@mailbox.sc.edu)

**Supplemental Table S1**. Number of gene targets for individual miRNAs identified by miRNet.

| miRNA | Gene Targets |
| --- | --- |
| miR-486-5p | 66 |
| let-7b-5p | 1215 |
| miR-142-3p | 388 |
| miR-221-3p | 367 |
| miR-29c-3p | 253 |
| miR-126-3p | 57 |
| let-7e-5p | 611 |
| miR-93-5p | 1219 |
| miR-7-5p | 577 |
| miR-146a-5p | 201 |
| miR-25-3p | 517 |
| miR-27b-3p | 420 |
| miR-92a-3p | 1406 |
| miR-29b-3p | 260 |
| **Total** | 7557 |

miRNet ([https://www.mirnet.ca](https://www.mirnet.ca/)) gene interaction data are collected from three well-annotated databases: miRTarBase v7.0, TarBase v7.0 and miRecords.


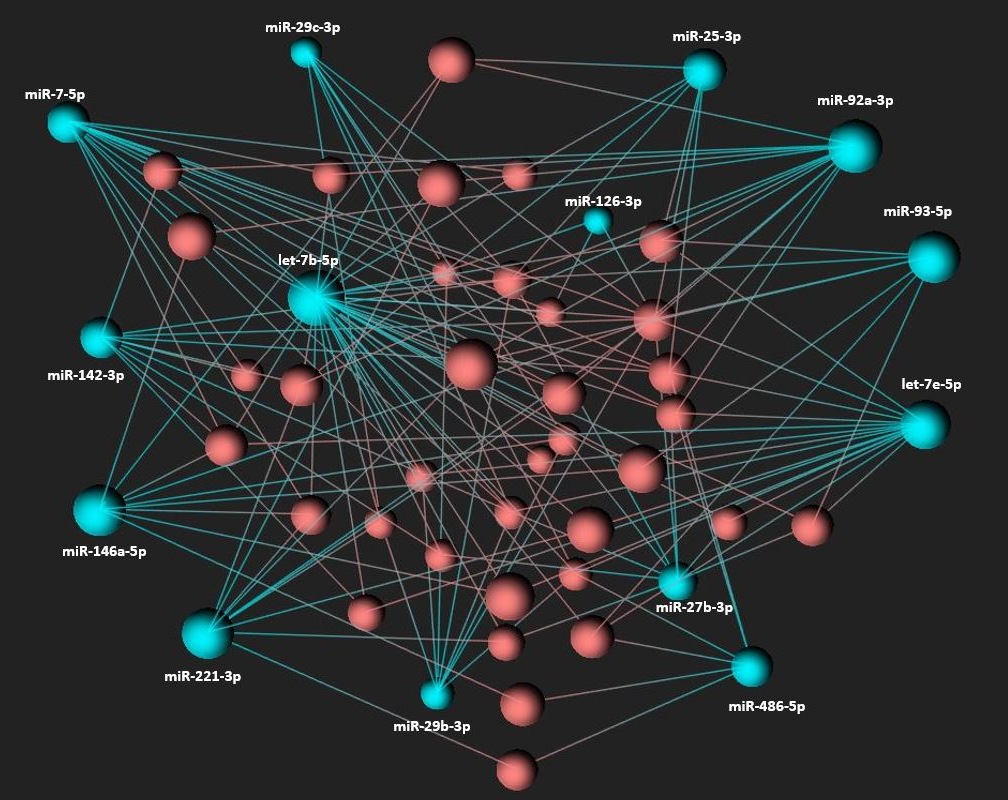


**Supplemental Figure S1**. Connectivity of the 14 miRNAs and their gene targets. Minimum network adjustment resulted in 47 nodes and 127 edges. MiRNAs are represented by the light blue nodes and genes are represented by the red nodes. Figure made using miRNet (<https://www.mirnet.ca>).
